# Long vs. short axial field-of-view PET scanners for brain imaging: a phantom study

**DOI:** 10.3389/fnume.2026.1856651

**Published:** 2026-06-19

**Authors:** Laura Providência, Valentina Turmacu, Philipp Mohr, Chris W. J. van der Weijden, Rudi A. J. O. Dierckx, Adriaan A. Lammertsma, Charalampos Tsoumpas

**Affiliations:** Department of Nuclear Medicine and Molecular Imaging, University Medical Center Groningen, University of Groningen, Groningen, Netherlands

**Keywords:** brain PET imaging, dose reduction, long axial field-of-view PET, sensitivity gain, short axial field-of-view PET

## Abstract

Long axial field-of-view (LAFOV) PET scanners offer substantially higher sensitivity compared to conventional short axial field-of-view (SAFOV) systems, potentially enabling reduced injected activity, shorter acquisition times, and improved signal-to-noise ratios in brain imaging. However, given the geometric differences between these systems, it is important to assess whether these differences impact image quality and quantitative accuracy before translating established SAFOV protocols to LAFOV systems. This study has two goals: (1) to compare image quality (IQ) metrics between a SAFOV PET scanner (Siemens Biograph Vision) and a LAFOV PET scanner (Siemens Biograph Vision Quadra) using a Hoffman brain phantom, and (2) to quantify the effective axial sensitivity gain provided by the LAFOV system for brain imaging.

**Methods:**

Two phantom experiments were performed: the first assessed IQ metrics and the influence of external background activity, while the second evaluated brain-specific sensitivity at different axial positions within the LAFOV scanner.

**Results:**

Overall, IQ metrics were highly comparable between scanners and were minimally affected by external background activity. Positioning the phantom at the first detector ring of the LAFOV scanner yielded a 1.2–1.4× sensitivity increase relative to the SAFOV system, whereas positioning it at the axial centre resulted in a sensitivity gain of up to 3.4×.

**Conclusion:**

These findings indicate that LAFOV PET systems maintain quantitative consistency with SAFOV scanners while offering higher sensitivity. This supports the development of optimized PET imaging protocols to reduce injected activity and radiation exposure, thereby improving safety and enabling broader inclusion of vulnerable populations in brain PET studies.

## 1 Introduction

Long axial field-of-view (LAFOV) PET scanners provide new opportunities for brain PET studies. Their higher sensitivity, which translates into larger signal-to-noise ratio (SNR) allows for shorter acquisition times, lower injected activity, delayed imaging protocols, and other protocol optimizations. For a fixed SNR comparable to SAFOV PET systems, the higher sensitivity of LAFOV PET systems can be used to reduce injected activity, acquisition time, or both (1). Reduction of the injected activity may be particularly advantageous in clinical scenarios requiring multiple PET tracers as part of a comprehensive diagnostic assessment [e.g., [${}^{18}$F]FDG combined with an amyloid or tau tracer (2)], as well as in longitudinal or follow-up studies where cumulative radiation exposure is a concern. Shorter acquisition times may be especially beneficial for patients with dementia or movement disorders, who may experience discomfort or have difficulty remaining still during prolonged scanning. Reduced scan duration can improve patient comfort, decrease motion-related artifacts, and enhance overall examination feasibility in vulnerable populations.

Although LAFOV PET offers substantially higher sensitivity, the translation of established SAFOV protocols to LAFOV systems requires careful evaluation. In particular, differences in scanner geometry may affect quantitative outcomes compared with established SAFOV protocols. For example, while the higher sensitivity of LAFOV systems is expected to improve image SNR, the larger acceptance angle also leads to increased scatter contributions and depth-of-interaction related effects associated with oblique lines of response (3, 4), which could potentially compromise quantification accuracy. Demonstrating consistency in standardized uptake values and overall image quality metrics (IQ) is therefore essential before such a transition can be made with confidence.

This study has two goals. First, to evaluate IQ metrics obtained from a Hoffman brain phantom acquired on both SAFOV and LAFOV PET systems, in order to assess potential differences between the two scanner types. Second, to quantify the brain-specific sensitivity gain of the LAFOV system across different axial positions within the scanner relative to a conventional SAFOV system.

## 2 Methods

### 2.1 Data acquisition

This study consisted of two separate phantom experiments designed to address two different questions. Dataset 1 was acquired to evaluate IQ metrics and to assess the potential influence of external background activity on LAFOV brain imaging compared with SAFOV imaging. Dataset 2 was designed to quantify the brain-specific sensitivity gain of the LAFOV system at different axial positions relative to the SAFOV system.

#### 2.1.1 Dataset 1

A Hoffman 3D brain phantom (5) was filled using a 1.5 L solution of ≈52 MBq 2-deoxy-2-[${}^{18}$F]fluoro-D-glucose ([${}^{18}$F]FDG), corresponding to an activity concentration of approximately 26 kBq/mL at the start of the PET acquisition. Two cylindrical ${}^{68}$Ge external sources (volumes of 9.5 and 9.6 L), with a combined activity of approximately 73 MBq, were positioned consecutively along the *z*-axis, in direct contact with each other and with the phantom to simulate activity originating from the body.

The phantom was first scanned on a Siemens Biograph Vision PET/CT scanner (axial field of view: 26 cm) (6) for 30 min without the external sources and subsequently for 40 min with the external source in place. Next (2 h and 10 min after the first Vision scan), the phantom was scanned in the first ring of a Siemens Biograph Vision Quadra PET/CT scanner (axial field of view: 106 cm) (7) for 80 min without the external sources and subsequently for 180 min with the external sources. The phantom was positioned in the first detector ring of the Quadra scanner to reflect common clinical LAFOV brain imaging configurations, where additional anatomical regions may also be included within the field of view, for example in whole-body acquisitions to assess brain in combination with other organs (e.g., brain-gut axis) or dynamic studies requiring image-derived input functions. All acquisition times were adjusted to have approximately the same total number of radioactive decay events as in the first scan, thereby taking into account the radioactive decay over time.

#### 2.1.2 Dataset 2

A second Hoffman 3D brain phantom experiment was performed using a 1.5 L solution containing 51 MBq of [${}^{18}$F]FDG, yielding an activity concentration of approximately 34 kBq/mL at the beginning of the first scan. The phantom was initially scanned for 10 min at the centre of the Siemens Biograph Vision scanner. It was then scanned in two positions on the Siemens Biograph Vision Quadra: (1) in the center of the first detector ring and (2) at the axial centre of the scanner, both also for 10 min. Identical acquisition durations were used across all scans to maintain comparable acquisition conditions. Because effective sensitivity (Equation 6) was normalized to both acquisition duration and activity at scan start, differences in radioactive decay between scans were explicitly accounted for in the calculation. The time interval between the Vision scan and the Quadra scans at the first detector ring and the axial centre was 48 min and 1 h 35 min, respectively.

### 2.2 Image reconstruction

All data (Datasets 1 and 2) were reconstructed using an ordered-subsets expectation maximization (OSEM) algorithm with point-spread function (PSF) modelling and time-of-flight (TOF), using 4 iterations and 5 subsets. The reconstructed image matrix was 440 × 440 × 645, resulting in voxel dimensions of 1.65 × 1.65 × 1.65 ${\mathrm{mm}}^{3}$. No post-reconstruction filtering was applied. Data were corrected for attenuation using a low-dose CT scan acquired on the respective PET/CT system. Additional corrections were applied for random coincidences, scattered radiation, dead time, and decay. The data acquired on the Siemens Biograph Vision Quadra scanner were reconstructed with maximum ring differences (MRD) of both 85 and 322. Image reconstructions were performed using e7tools, a prototype research software package from Siemens Healthineers.

### 2.3 Image analysis

**Figure 1:**
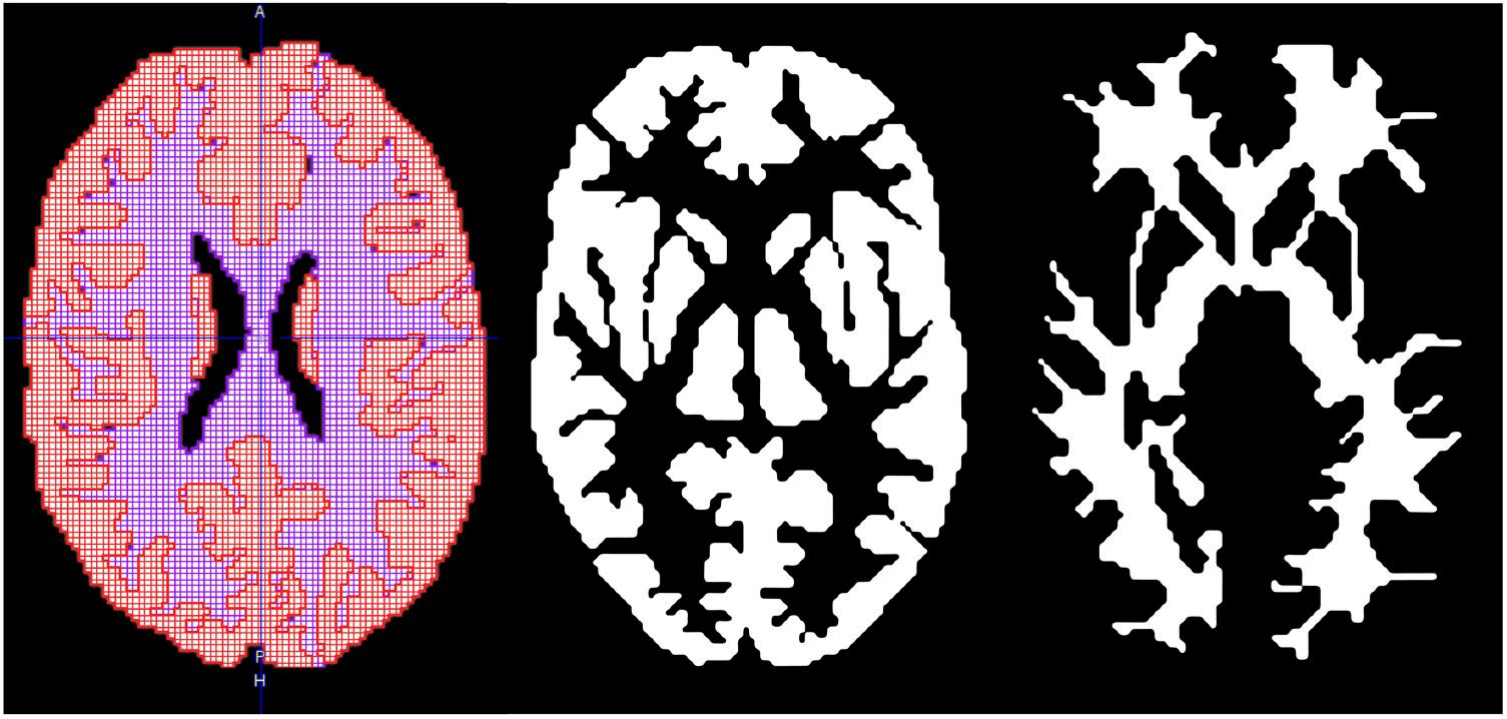
Grey and white matter VOIs delineations. Left: whole brain grey (red) and white (blue) matter masks generated in PMOD from the Hoffman phantom DRO. Middle: whole brain grey matter binary mask. Right: whole brain white matter binary mask.

The digital reference object (DRO) for the Hoffman phantom (28) was used to delineate grey matter (GM) and white matter (WM) volumes of interest (VOIs). Additional VOIs covering the whole brain as well as the left and right hemispheres were also generated. To reduce partial volume effects in subsequent image quality metrics, eroded versions of all anatomical VOIs (grey matter, white matter, and their hemispheric subdivisions) were generated using spherical kernels with radii of 2, 3, and 4 voxels. Five spherical VOIs were placed in white-matter regions following the approach described in (8). Furthermore, a 2.59 mL spherical VOI was positioned in the solid plastic region of the phantom (region with zero uptake in between regions of higher uptake) to simulate a low uptake midbrain region (VOI_cold_), as described in (9). All VOI definitions were performed in PMOD (version 4.1, PMOD Technologies LLC, Zurich, Switzerland), and the resulting individual VOIs were exported as NIfTI binary mask files for subsequent analysis (Figure 1) in Python (version 3.13.5).

To ensure consistent spatial alignment, all PET images were registered to a reference PET scan. Image registration was performed using the open-source NiftyReg software package (10), specifically the *Aladin* algorithm constrained to rigid transformation. For Dataset 1, all images were registered to the Quadra acquisition without the external source; for Dataset 2, all images were registered to the Quadra acquisition in the first ring. The digital reference object DRO of the Hoffman phantom was also registered to the appropriate reference scan for each dataset, and the corresponding affine transformation matrix from each registration was saved. These matrices were then applied to the DRO binary masks using NiftyReg’s *RegTransform* tool to bring the VOIs into PET image space. Because the DRO has a matrix size of 256 × 256, the transformed masks were resliced to match the PET image matrix (440 × 440). All image processing steps were implemented in Python using the *Nipype* software package (11). The registered masks were visually inspected to confirm accurate alignment with the PET images.

### 2.4 Quantification of image quality

Indicators of image quality were calculated as described in (8) using Dataset 1. The values were calculated for Vision, Quadra first ring (MRD 85 and 322) and Quadra centre (MRD 85 and 322). In the equations, “Dose-calibrator activity concentration” corresponds to the activity concentration of the stock solution (9), calculated by measuring the syringe activity (kBq) and dividing it by the volume of water used to fill the phantom. The evaluated IQ metrics are shown in Equations 1–5:

1. Grey matter recovery coefficients (GMRC), calculated as:
  $$\text{GMRC}=\frac{\text{Mean activity concentration in GM VOI}}{\text{Dose-calibrator activity concentration}}$$ (1)
2. Contrast that, for the Hoffman phantom, should ideally be 4:
  $$\text{Contrast}=\frac{\text{Mean activity concentration in GM VOI}}{\text{Mean activity concentration in WM VOI}}$$ (2)
3. Coefficient of variation (COV%), used as an indicator of image homogeneity in a uniform background, and calculated for the five spherical VOIs placed in the white matter:
  $$\begin{array}{rl} & \text{COV}(\%)\\ & =\frac{\text{Standard deviation of activity concentration in }{\text{VOI}}_{\text{sphere}}}{\text{Mean activity concentration in }{\text{VOI}}_{\text{sphere}}}\\ & \;\times 100 \end{array}$$ (3)
4. Left to right hemisphere RC ratio, which should ideally be 1, to assess symmetrical performance of software and hardware of PET scanners:
  $${\mathrm{LR}}_{\text{ratio}}=\frac{\text{GMRC of right hemisphere}}{\text{GMRC of left hemisphere}}$$ (4)
5. Cold spot recovery using VOI_cold_, calculated as:
  $$\text{Cold-spot RC}=\frac{\text{Mean activity concentration in }{\text{VOI}}_{\text{cold}}}{\text{Dose-calibrator activity concentration}}$$ (5)
6. Ratio between image-derived activity concentration and the dose-calibrator activity. The image-derived activity concentration was calculated as described by (8). Briefly, the DRO was smoothed using an 8 mm full width at half maximum Gaussian filter. A binary mask was then generated by selecting voxels with intensity values greater than 0.98. This mask was subsequently applied to determine the mean image-derived activity concentration. This measurement is used to assess calibration between the dose calibrator and the PET system, and its value should fall between 0.9 and 1.1 according to EARL criteria (12).
7. Effective image resolution, as described in (8).

GMRC and contrast were calculated both with the full VOIs as well as with the eroded VOIs.

### 2.5 Effective axial sensitivity for brain imaging

The second dataset was used to calculate the effective axial sensitivity ($S_{\mathrm{eff}}$) when imaging the brain placed at the centre of the Vision, Quadra first ring and Quadra centre. In this context, sensitivity does not refer to the intrinsic NEMA sensitivity, but rather to the object and protocol dependent sensitivity experienced when imaging a Hoffman brain phantom, which depends on the scanner and axial position within the field of view. This value was calculated with the following equation:

$$S_{\mathrm{eff}}=\frac{T}{A_{0}\;\Delta t}$$ (6)

Where T is the number of true events, Δt the duration of the scan, and $A_{0}$ the activity on the phantom at the beginning of the scan.

## 3 Results

### 3.1 Image quality metrics

**Figure 2:**
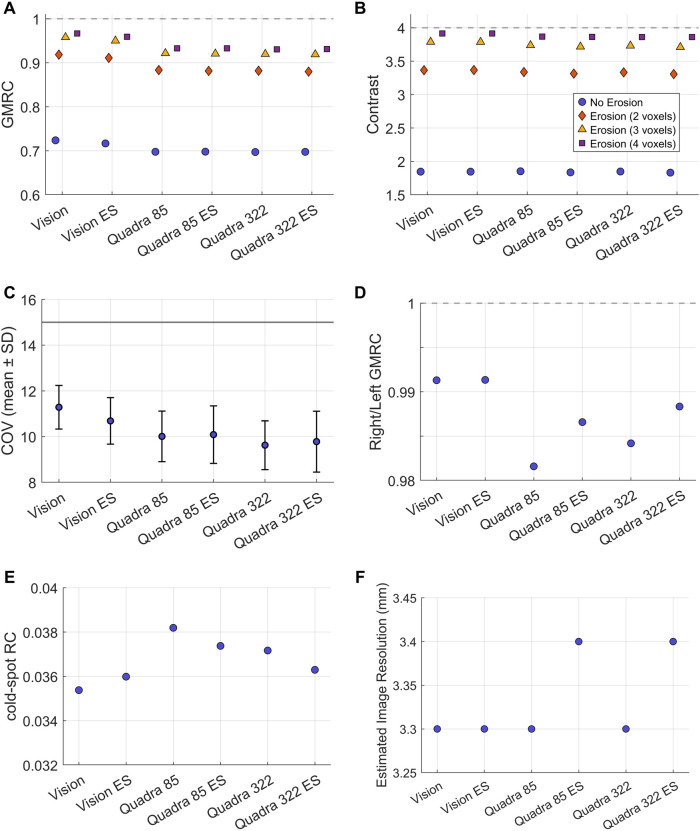
Image quality metrics calculated for Vision and Quadra PET scanners. **(A)** Grey matter recovery coefficient (GMRC), **(B)** contrast, **(C)** Coefficient of variation (COV): dots and error bars represent the mean and standard deviation (SD) calculated from five spherical VOIs placed in the white matter, **(D)** right-to-left hemisphere recovery coefficient, **(E)** cold-spot recovery, and **(F)** estimated image resolution. The dashed grey lines indicate the ideal values. The full grey line in subfigure **(C)** indicates the acceptance criteria proposed by (8). ES: external source; 85 and 322 indicate the maximum ring difference used during image reconstruction.

Results for the image quality metrics are presented in Figure 2. For all scanners and acquisition set-ups, erosion of the VOIs led to improved GMRC and contrast, with an erosion of four voxels yielding the best results (Figures 2A,B). GMRC values were slightly higher for the Vision scanner (0.96 and 0.97 without and with external sources, respectively) compared with the Quadra scanner (0.93 for all set-ups). Contrast values were highly consistent across scanners and acquisition conditions, with a value of 3.9 for all configurations.

The average COV values remained largely stable across scanners and acquisition set-ups (Figure 2C). The highest COV values were observed for Vision without (11.3%) and with (10.7%) external source. These were followed by the Quadra scanner reconstructed with MRD85, with average COV values of 10.1% (with external source) and 10.0% (without external source). The lowest COV values were obtained for Quadra MRD322 reconstruction, with mean values of 9.6% and 9.8% for acquisitions without and with external sources, respectively.

The left-to-right hemisphere RC was stable across scanners and acquisition set-ups, ranging from 0.98 to 0.99 (Figure 2D). The cold-spot RC was identical across all scanners and acquisition conditions, with a constant value of 0.04 (Figure 2E). The estimated image resolution also showed minimal variation, ranging from 3.4 mm for the Quadra acquisitions with external sources to 3.3 mm for all other configurations (Figure 2F).

**Figure 3:**
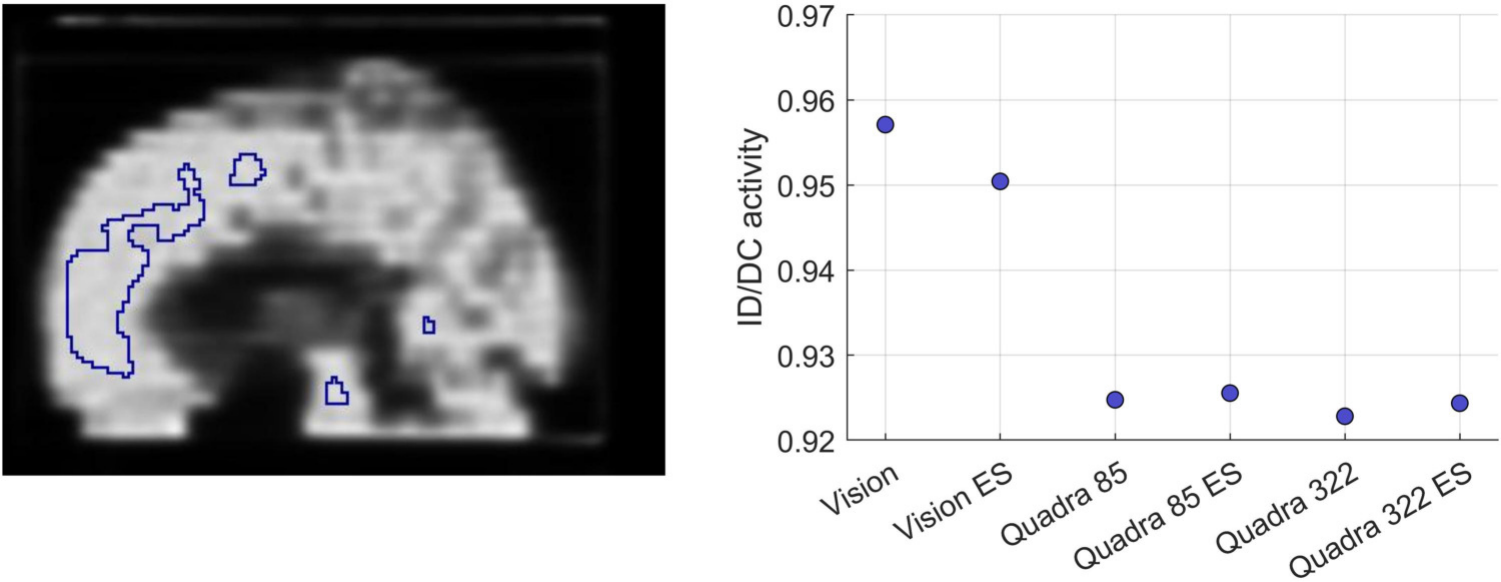
**Left:** VOI used for calculation of image-derived activity concentration. **Right:** Ratio between image-derived (ID) and the dose-calibrator (DC) activity.

The VOI used to calculate the ratio between image-derived activity concentration and dose-calibrator activity concentration is shown in Figure 3 (left). The ratio was 0.96 and 0.95 for Vision acquisitions, and 0.92 and 0.93 for Quadra acquisitions (Figure 3, right), meeting the acceptable level of error (±10%) based on EARL criteria (12).

### 3.2 Effective axial sensitivity for brain imaging

**Figure 4:**
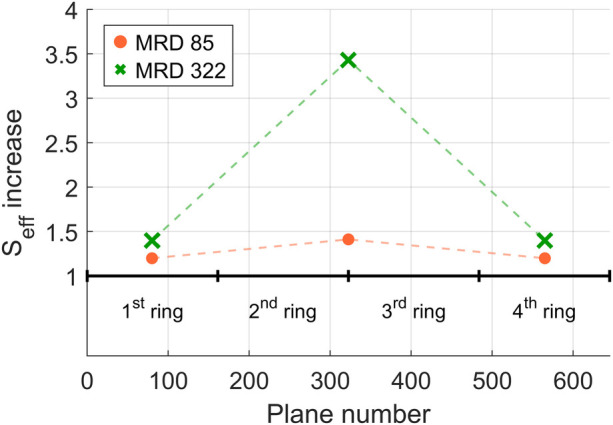
Ratio of effective axial sensitivity ($S_{\mathrm{eff}}$) measured with a Hoffman brain phantom in a LAFOV PET relative to SAFOV. The values shown for the fourth Quadra ring replicate those of the first ring, assuming axial scanner symmetry. Results are shown for maximum ring differences (MRD) of 85 (orange circles) and 322 (green crosses).

When the Hoffman phantom was positioned at the first Quadra ring, $S_{\mathrm{eff}}$ was 1.2-fold (MRD85) and 1.4-fold (MRD322) higher than that of the Vision. When positioned at the centre of the Quadra scanner, $S_{\mathrm{eff}}$ increased to 1.4-fold (MRD85) and 3.4-fold (MRD322) relative to the Vision (Figure 4).

## 4 Discussion

This study evaluated the differences in IQ metrics obtained from scanning a Hoffman brain phantom in both a SAFOV (Biograph Vision) and a LAFOV (Biograph Vision Quadra) PET scanner, including the impact of external sources on these metrics. The effective Hoffman phantom axial sensitivity gain achieved at different axial positions within the LAFOV system was also quantified and compared with that of the SAFOV system. Overall, IQ metrics were consistent across scanners, reconstruction settings (MRD 85 and 322), and the presence or absence of an external source. While placing the brain phantom in the first ring of the LAFOV scanner resulted in an effective sensitivity gain of approximately 40% relative to Vision, positioning the phantom at the axial centre of the LAFOV scanner led to a larger gain of up to 3.4.

The Vision and Quadra scanners share the same detector technology and differ primarily in axial coverage and, consequently, system sensitivity. While major differences in image quality metrics are not expected, the larger field of view raises the question of whether increased scatter contributions and oblique lines of response could potentially affect quantitative accuracy. Overall, differences in IQ metrics between Vision and Quadra acquisitions were minimal. The largest discrepancy was observed for GMRC, which was approximately 3% lower for Quadra (GMRC = 0.93) compared with Vision (GMRC = 0.97). These findings are consistent with the image-derived activity concentrations measured for both scanners. Specifically, the image-derived activity from Quadra acquisitions corresponded to 0.93 of the dose-calibrator activity concentration, whereas the Vision scans were 0.96 (without external source) and 0.95 (with external source) of the dose-calibrator activity concentration. Importantly, when GMRC is calculated by dividing the grey matter activity concentration by the image-derived activity rather than by the dose-calibrator activity, as performed by (9), the differences between Quadra and Vision are eliminated (all GMRC ≈ 1.01). With this approach, the GMRC values fall within the same range reported in (9) for GMRC (eroded VOI) of the non-harmonized PET images. The lower image-derived to dose calibrator-derived activity ratio observed for Quadra may be attributable to calibration differences between the systems. Such differences are expected to primarily affect GMRC values rather than reflect intrinsic differences in image quality between scanners.

The impact of external sources on LAFOV acquisitions was negligible. GMRC, contrast, cold-spot recovery, and the ratio between image-derived and dose-calibrator activity concentrations remained unchanged. Inclusion of external sources led to only minor increases in mean COV (0.2% for MRD322) and a marginal increase in estimated image resolution (0.1 mm). These findings indicate that the potentially higher scatter contribution in LAFOV PET scanners does not significantly affect Hoffman phantom IQ metrics. Similarly, the presence of an external source did not affect IQ metrics in the Vision acquisitions.

The effective sensitivity gain achieved by positioning the Hoffman phantom at the middle of the first ring of the Quadra relative to the Vision was approximately 20% and 40% for MRD85 and MRD322, respectively. This magnitude is lower than the 5–10× sensitivity increases commonly reported for LAFOV PET systems (13), which was expected as the brain is already fully encompassed within the SAFOV and therefore does not benefit as much from extended axial coverage as multi-bed acquisitions. This observation is consistent with similar COV values obtained for Vision and Quadra acquisitions in this configuration (Figure 3). Importantly, all COV values remained well within the acceptable 15% threshold suggested in the literature (8), even though no harmonization was performed. MRD 85 is clinically suggested when uniform sensitivity is essential, while MRD 322 provides maximum possible sensitivity and reduced image noise. Furthermore, previous studies have shown that the inclusion of highly oblique lines of response in MRD 322 may lead to a slight degradation in axial spatial resolution, particularly near the centre of the axial field of view (2 to 3 mm) (14). If this resolution degradation is not a concern, the choice of MRD 322, particularly for brain imaging, appears to provide more advantages independent of where the head is positioned within the scanner.

Positioning the phantom at the axial centre of the LAFOV scanner resulted in a 3.4-fold increase in effective sensitivity. This increase is consistent with theoretical predictions (15), which estimate a 2.5- to 3.5-fold sensitivity gain for LAFOV systems compared with SAFOV systems in single organ imaging. Taking this into account, it is theoretically possible to reduce the injected activity and/or the acquisition time while maintaining comparable image quality. This may be particularly relevant for clinical brain PET applications where reduction of radiation exposure is desirable, such as longitudinal studies, paediatric imaging, or research protocols involving healthy volunteers. Table 1 summarizes selected brain PET tracers, current guideline-recommended protocols, and theoretical low-dose protocols derived from the estimated 3.4-fold sensitivity gain observed at the axial centre of the LAFOV scanner. The effective doses presented in Table 1 were estimated by combining the reduced PET tracer activity with the effective dose contribution from a previously proposed ultra-low-dose CT protocol (29). In this table, the increased sensitivity was applied exclusively to reduce the injected activity. Alternatively, the same sensitivity gain could be used to reduce the acquisition time by a factor of approximately 3.4 while maintaining the original administered activity, following validation of the appropriate post-injection time window for scanning. A proportional reduction of both parameters could also be implemented. This makes PET imaging accessible to a broader range of patient groups, including children, healthy subjects, pregnant women, or intensive care patients. Importantly, these reductions are theoretical estimates derived from phantom-based sensitivity measurements. Before clinical implementation, validation in patient studies is required to confirm preservation of diagnostic image quality and quantitative accuracy. Alternatively, the injected activity and acquisition time can be maintained to achieve improved image quality, enabling more robust kinetic modelling approaches such methods requiring high-temporal resolution dynamic imaging (16), parametric imaging (17), or reference tissue models (18).

**Table 1:** Overview of selected PET tracers for clinical brain imaging, including current guideline recommended administered activities and suggested activity reductions based on a 3.4-fold increase in scanner sensitivity.

Tracer	Act. rec.	Act. sugg.	ED coeff.	ED total	Dur.	Source
	(MBq)	(MBq)	(mSv/MBq)	(mSv)	(min)	
${[}^{18}$F]FDG	125–250	37–74	0.019	0.8–1.5	10–15	NIC (19)
${[}^{18}$F]FDOPA	185	54	0.025	1.4	20	FDA (20)
${[}^{18}$F]Florbetapir	370	109	0.0186	2.1	10	SNMMI/EANM (21)
${[}^{18}$F]Flutemetamol	185	54	0.032	1.8	20	SNMMI/EANM, EMA (21)
${[}^{18}$F]Florbetaben	300	88	0.0193	1.8	20	SNMMI/EANM (21)
${[}^{18}$F]Fluorotaucipir	185	54	0.025	1.4	20	(22)

Act. rec, current recommended administered activity; Act. sug, suggested activity reductions based on a 3.4-fold increase in scanner sensitivity; ED, tracer-specific effective dose coefficient; ED total, effective dose from PET and ultra -low-dose CT; Dur, current recommended acquisition time; NIC, EANM Neuro-Imaging Committee; EANM, European Association of Nuclear Medicine; SNMMI, Society of Nuclear Medicine and Molecular Imaging. The tracer-specific effective dose coefficients were obtained from: ICRP 106 for [${}^{18}$F]FDG and [${}^{18}$F]FDOPA (23), (24) for [${}^{18}$F]Fluorotaucipir, (25) for [${}^{18}$F]Florbetaben, (26) for [${}^{18}$F]Flutemetamol, and (27) for [${}^{18}$F]Fluorotaucipir.

This study has a number of limitations. IQ metrics were assessed only with the phantom positioned in the first ring of the LAFOV scanner; however, these results are expected to translate to other axial positions with higher sensitivity, where improved COV values are expected. Similarly, Hoffman phantom effective sensitivity was evaluated at a limited number of axial positions; a more comprehensive assessment, including a position at the axial edges of the scanner and at the centre of the second ring, would provide a more complete sensitivity profile. The two evaluated positions were selected to represent a clinically relevant positioning configuration (first detector ring) and a configuration for which sensitivity is maximised for brain imaging (axial centre). These represent the two extreme limits, other positions would be within these two limits. Therefore, we do not expect inclusion of additional positions to change the main conclusion of the study. In addition, all phantom experiments were based on a single measurement and the scans were not repeated, which limits the assessment of measurement variability and reproducibility. Finally, the Hoffman phantom represents a simplified model of the human brain and does not capture the full anatomical and functional complexity of in vivo tracer uptake. Therefore, confirmation of these findings in human subjects may be required for certain clinical questions.

Despite these limitations, the results demonstrate a high degree of quantitative consistency between SAFOV and LAFOV PET systems. This supports the feasibility of translating established SAFOV brain PET protocols to LAFOV PET scanners and highlights the potential for reduced injected activity or shorter scan durations when the brain is positioned near the axial centre of the LAFOV scanner.

## 5 Conclusion

Hoffman phantom image quality metrics are highly comparable between SAFOV and LAFOV PET systems. To fully exploit the higher sensitivity of LAFOV PET scanners, subjects should be positioned with the head near the axial centre of the field of view, where up to a 3.4-fold increase in sensitivity relative to SAFOV PET scanners can be achieved.

## Data Availability

The raw data supporting the conclusions of this article will be made available by the authors upon request, without undue reservation.
